# A change of direction for family policy in Italy: some reflections on the general family allowance (GFA)

**DOI:** 10.1186/s41118-023-00193-x

**Published:** 2023-05-22

**Authors:** Gianpiero Dalla-Zuanna, Peter F. McDonald

**Affiliations:** 1 Department of Statistical Sciences, University of Padua, Padua, Italy; 2 School of Population and Global Health, Melbourne University, Parkville, Australia

**Keywords:** Family allowances, Low fertility, Italy, Developed countries

## Abstract

We present and discuss the General Family Allowance (GFA), in Italian: *Assegno Unico Universale*, a measure that the Italian Government and Parliament have put in place from March 2022 addressing the persistent low fertility in Italy. The GFA modernizes monetary transfers in favor of families with children in Italy, covering large groups of families that were previously excluded from full benefits. Even if the aim of the GFA is to support fertility rather than to alleviate child poverty, it is likely that this measure will help to reduce poverty, especially for families with children previously excluded from significant cash contributions, such as recently resident foreigners and the unemployed. In addition, as GFA amounts are modest for wealthier couples, its potential effect on fertility—if there will be any—should be limited to couples with modest incomes. The GFA is also compared with the different systems of monetary transfers in favor of families with children of developed countries.

## Introduction

The Law n. 46 of 1st April 2021, established in Italy the General Family Allowance—GFA (in Italian *Assegno Unico Universale*), with the aim of “promoting the birth rate, supporting parenthood and promoting employment, especially for women” (article 1). The GFA is the first step towards a distancing from past policies related to family transfers. These policies were a mix of different kinds of measures: those aiming at sustaining the fertility level and those aiming at averting poverty among families with children. It has been found that they were ineffective in meeting both goals. The introduction of the GFA, together with the Universal Basic Income—UBI (in Italian *Reddito di Cittadinanza*, Law n. 26 of 28th March 2019) was also a move to separate fertility-support policies from income-support policies. The fact that the middle class (which is not included in the traditional measures addressing poverty) benefits from the GFA is another sign of this policy direction. With this change, Italy is following other European countries, especially Germany and France, where child allowances are not designed to address children poverty.

However, in accordance with the previous family allowances design, there is still an important component of the GFA that is income-tested contrasting with a “pure” pronatalist policy, especially compared to the German approach, where the *Kindergeld* is not commensurate with the income or wealth of the parents. Considering this and the debate and the studies that have preceded and followed the introduction of the GFA, we also consider its possible effects on the reduction of child poverty. Trends towards lower fertility and higher child poverty have prevailed in Italy for many years, deriving from deep-rooted social, cultural and economic causes that are not easy to remove (for lowest low fertility, see Billari, 2008, Caldwell & Schindlmayr, 2003, Dalla Zuanna & Micheli, 2004, Livi-Bacci, 2004, Tanturri, 2016; for child poverty, see Baldini et al., 2018, Natali & Saraceno, 2017, Saraceno et al., 2020).

The GFA has been so important in the framework of Italian family policies, that it is worth describing in detail its meaning, objectives, elements of break and continuity with respect to previous policies, and its position in the framework of family policies of the developed countries. This provides a means of assessing the likely effectiveness of this policy especially in terms of increasing the fertility rate.

In this article, after discussing the general context of family allowances in developed countries, considering both horizontal and vertical equity, we briefly summarize the persistent low fertility and the growing poverty of minors in Italy. Then, we describe the GFA. It is too early to measure the full impact of GFA on fertility and its effect in reducing poverty as the measure was fully effective only from 1st March 2022. However, in the final parts, we describe some findings from modeling of the impact and draw some hypotheses in this regard.

### The family allowance policies of the developed world

Here, the term, family allowance, will be used to describe per child, cash payments made by governments across the lifetime of a child. The central rationale for governments to pay a family allowance to parents is that children are a social good. The argument is that, fundamentally, children are tomorrow’s citizens and tomorrow’s workers and, as such, support of families with children is an investment in the country’s future. This was recognised in many countries in the latter half of the nineteenth century with the introduction of universal education for children funded by the state and the associated child labour laws. Today, education of all children is a core feature of the development plans of virtually every developing country.

In the context of children as a social good, financial support for families with children derived originally from the principle of horizontal equity. Horizontal equity is the recognition by government in the tax-transfer system that those raising the next generation of children face additional costs compared with those having the same family income but are not raising children. Family allowances aim to compensate for this additional cost at least in part. Of course, there are other ways that governments can compensate for the costs of children including support for services such as education and childcare, differential tax treatment or support in the form of cash or paid parental leave provided at the time of each birth. However, even in the most generous of welfare states, upwards of 50% of the costs of children are met by parents (National Transfer Accounts Data Sheet, 2016) and this is an inevitability given public fiscal constraints.

Family allowances have their origins in welfare state regimes: France 1932, but only for those employed; Australia 1941, universal; United Kingdom 1946, universal; Nordic countries late 1940s, universal; Federal Republic of Germany 1949, but only for those paying social insurance. These welfare state origins placed family allowances in a context of vertical equity, the principal that the state should provide increasingly higher levels of support to families as income levels fall. While a standard cash payment made in respect of all children is of greater value to poor families than to rich families, vertical equity tends to imply a steeper slope to the level of family allowance received as incomes rise. Consequently, the question has arisen: why should governments spend scarce fiscal resources on children living in rich families? The power of this question has led to means-testing of family allowances in many countries in the past 40 years. The design of the new allowance in Italy, universal but income tested, is in keeping with this history of family allowances.

The origins of family allowance payments are also associated with the emergence of low fertility rates in the 1930s. Thus, there was an overtone of pronatalism associated with the introduction of family allowances. However, there are cogent arguments that fertility today, in the context of the employment of both parents, is influenced by benefits that address the more immediate costs of a birth, essentially the loss of parental income and the disruption to carers of absences from the labour force (Billingsley et al., 2022).

Today, family allowances across countries come in a variety of shapes and forms including universal flat payments, universal means-tested payments, payments where a variable percentage of wealthier families do not receive the payment, payments where the age range of eligible children varies, payments that vary according to the parity of the child including the exclusion of children of particular parities (first child, third or higher order children). There is also variation in which parent of the child receives the payment. A global study is provided by ODI/UNICEF (2020).

The ODI/UNICEF report favours a universal family allowance paid in respect of every child, including those in well-off families (recognising horizontal equity) but, with its focus on the alleviation of child poverty, the report also favours higher payments to children in low-income families (recognising vertical equity). Horizontal and vertical equity can be pursued with separate policies, as happens, for example, in Germany, and this way of acting is certainly more efficient, easier to convey to public opinion and simpler to subject to impact assessment. However, even a measure that aims to combine the two types of equity, if well-designed, can allow for progress in both directions, especially if adopted to replace largely deficient measures. As we will see in the next parts, this is exactly the structure of the GFA. The GFA is a simple system not complicated by exclusions related to the employment of parents or the parity of the child. When viewed from the perspective of the child (not responsible for its parity or the employment of its parents), this is an ideal approach. The means testing of the GFA at higher incomes implies increases to effective marginal tax rates, but this is offset to some extent by the additional payments, where both parents are employed. In addition, at higher income levels, modest increases to effective marginal tax rates are unlikely to be a work disincentive.

### Low fertility and growing poverty of minors in Italy

**Fig. 1:**
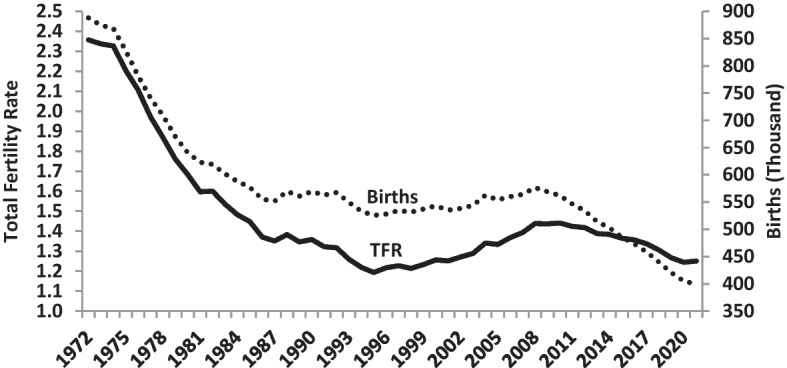
Total Fertility Rate (TFR) and the number of births in Italy, 1972–2021. Source: Istat—The National Statistics Institute

In Italy, in 1984 for the first time, the TFR fell to 1.5 births per woman, and it has remained below this level ever since. Births also decreased, due both to the persistently low fertility and the progressive exit from the childbearing ages of the baby-boomer generation born between 1955 and 1975, who had sustained the number of births up to the first years of the twenty-first century. In 2022, the number of births in modern Italy fell below 400,000 for the first time, less than half the number 60 years earlier (Fig. 1). Despite this, in 2021, Italy had 2.7 million more inhabitants than in 1981, due to the relatively high number of boomers in the childbearing age from 1981 to 2010, the substantial increase in survival (e_0_ rose from 74.4 in 1981 to 82.4 in 2021) and immigration which was particularly strong in the first decade of the new century. In 2021, the number of people born in another country who were living in Italy was 6.7 million, while, in 1981, their number had been negligible (Strozza, 2022).

However, despite immigration, 40 years of lowest low fertility and increasing survival have led to rapid ageing of the Italian population (Table 1). The number of young people has fallen dramatically; those aged under-15 numbered 12.2 million in 1981 but only 7.6 million in 2021. According to the National Statistics Institute (Istat)—which forecasts an average of 1.4 children per woman for the next 20 years, a further growth of e_0_ to 85.5 by 2041 and an annual net migration balance of + 140,000—the number aged less than 15 will fall further to just 6.4 million in 2041, half the level of 60 years earlier. The 15–39 age group has also shrunk and is expected to shrink further, while the number of adults aged 40–64—which increased between 1981 and 2021—will drop sharply over the next two decades as the baby-boom generation gets older. As a result, the percentage aged 65 years and over will have increased from 13% in 1981 to 33% in 2041.

**Table 1:** Population in Italy by age class. 1981, 2001 and 2021 (effective), 2041 (forecasted)

	Million				Column %			
	1981	2001	2021	*2041*	1981	2001	2021	2041
0–14	12.1	8.1	7.6	*6.4*	21	14	13	*11*
15–39	20.2	19.8	15.6	*13.8*	36	35	26	*25*
40–64	16.8	18.4	22.1	*17.3*	30	32	37	*38*
65 +	7.5	10.6	13.9	*18.7*	13	19	24	*33*
Total	56.5	57.0	59.2	*56.2*	100	100	100	*100*

Sources: Istat data and projections published by Strozza (2022) (2001–2041) and our elaborations (1981)

The rapid aging of the Italian population, its reduction (from 60.3 million on 1.1.2014 to 59.0 million on 1.1.2022) and the collapse in the number of births (from 580,000 in 2008 to 405,000 in 2020) have contributed to increasing the awareness in public opinion of the need to implement more effective policies, including monetary ones, in favor of couples wishing to have an (extra) child, overcoming the last hesitations evoking the policies of the fascist regime, thus pushing the policy to design and implement horizontal equity interventions.

**Fig. 2:**
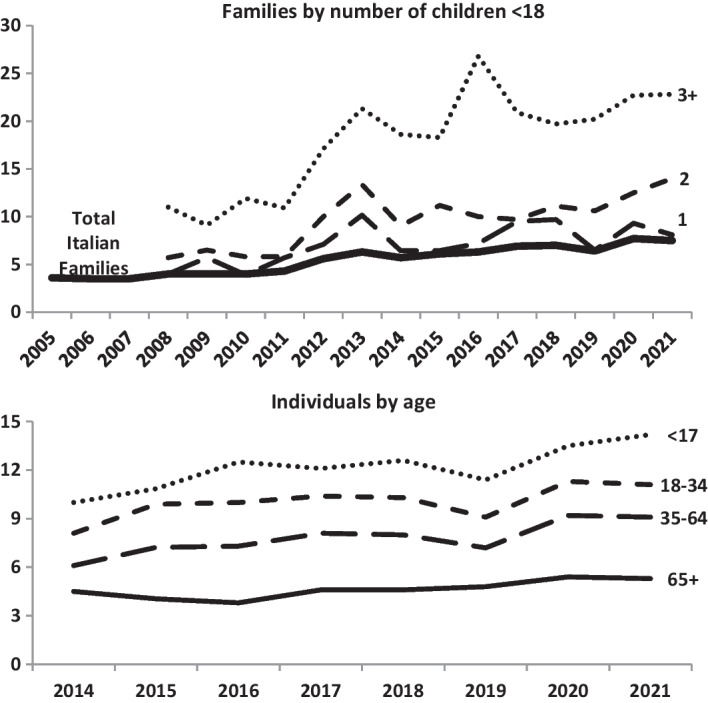
Percentage in absolute poverty in Italy*. *Families with a monthly expenditure equal to or lower than the value of the absolute poverty threshold are classified as absolutely poor, i.e., the minimum expenditure necessary to acquire basic necessities and services included in the basket of absolute poverty. This threshold differs in size and composition by age of the family members, geographical location and type of municipality of residence, taking into account differences in purchasing power (Istat, 2009). Source: Istat—The National Statistics Institute

Since 2005, Istat has published a report on poverty in Italy every year, using the data from the annual survey on consumption. Since 2008, the proportion of families in absolute poverty by number of minor children has been published, and the proportion of poor individuals by age has been available since 2014 (Fig. 2).

Poverty levels for individuals and families increased to 2019 when, for the first time in Italy, a heavily funded measure against poverty (the UBI) was established. However, the growth of poverty resumed in the following 2 years due largely to the sudden growth of unemployment related to COVID-19, mainly for marginal jobs, often the only option for poor people.

Figure 2 also shows that poverty is and has been higher among families with children as opposed to all families, and the level of poverty increases as the number of children in the family increases. At the individual level, poverty is highest for children compared with older persons. In contrast, the percentage of older people is low and has not changed across time. In 2020 and 2021, one in four families with 3 or more children had great difficulty in providing for basic expenses, such as food, clothing, gas, electricity and water. However, the level of absolute poverty has grown significantly also for families with two children: from 6% in 2008 to 14% in 2021.

These data on growing child poverty in Italy have also had a significant impact on public opinion, contributing to increasing the demand for effective vertical equity interventions aimed at families with children. In addition, because—for the reasons we will mention—UBI has not proved to be particularly effective for this purpose.

### Inequalities and confusion before GFA

Prior to the GFA, in 2019 and across a range of programs, the Italian state disbursed 14 billion euros (0.8% of GDP) in direct monetary transfers to families with children. The two main programs were family allowances for employees and tax deductions for income recipients, both of an amount inversely proportional to income. These two measures were conditioned by the employment and income status of the parents: an employee who became unemployed—losing both job and income—lost the right to both family allowances and tax deductions, and self-employed workers and freelancers, very numerous in Italy, were not entitled to family allowances.

Over time, there have also been measures against poverty of children, the main ones being the law 448/1998 for poor families with 3 or more children and the aforementioned UBI. Law 448/1998 helped large families and increased births and limited abortions among poor women with 2 or more children (Billari et al., 2005). However, Law 484/1998 was not able to curb the increase of poverty in this group, at least during the years covered by Fig. 2, as the amount (at the end of 2021) was only 145€ a month for each family.

The UBI came into force on 1st April 2019, with substantial funding and assisted many people in difficulty. Istat (2022a, chapter 3) has calculated that, mainly due to the UBI, one million people have been saved from absolute poverty during the pandemic years, 2020–21. However, as far as the poverty of children is concerned, the hUBI as two serious defects: it grows only slightly in proportion to the number of family members and can only be assigned to non-EU citizens who have lived in Italy for at least 10 years. For these reasons, as Fig. 2 shows, in 2019 compared to 2018, the reduction in poverty was non-existent among families with 3 or more children, among whom non-EU citizens are overrepresented. Among families, where all members were non-EU citizens, absolute poverty decreased between 2018 and 2019 (from 27.8 to 24.4%) but remained five times higher than among families with all Italian members, which dropped from 5.3 to 4.9%.

Tax bonuses for families with children, variously structured, have been established and then abolished by the various governments that have followed one another in Italy. In general, however, these benefits have always been a matter of symbolic sums, insufficient to affect poverty or fertility.

Finally, Regions and Municipalities also distribute monetary transfers. In general, these are modest amounts and mostly concentrated in the first part of a child’s life. Exceptions are the Provinces and the Autonomous Regions which have greatest resources. It is no coincidence that in the Alpine Autonomous Provinces of Trento and Bolzano/Bozen (Northeast Italy), fertility is higher than the Italian average (in 2021: 1.42 Trento, 1.71 Bolzano/Bozen, 1.25 Italy). It has been shown that this higher fertility is associated with monetary transfers, as well as high per capita income and other policies towards families (Dalla-Zuanna et al., 2020; Matsiuk, 2022). Moreover, a substantial transfer to couples with medium–low income at the birth of a child of order 2 or more implemented by the Friuli-Venezia Giulia Region in 2000–03 increased the probability of having a new birth and decreased the probability of having an abortion for low-income women with at least one child already born (Boccuzzo et al., 2008). Several local transfers have continued after the introduction of GFA.

### The structure of GFA

The law establishing GFA had already been proposed by the Progressive Party (*Partito Democratico*) in 2014. After a long process, it obtained the final go-ahead from the Italian Senate on 30th March 2021, approved by all members without any negative vote (Law 46/2021). The definitive version (with detailed indication of the amounts and methods of request and disbursement) was then established by the Legislative Decree 230 of 21st December 2021. GFA started in a complete way from 1st March 2022. GFA absorbs the national-level, monetary transfers described in the previous part, but the law has allocated an additional six billion euro, reaching a total of 20 billion euro which is equivalent to around 1% of Italian GDP. These expenditures—like the individual payments for families—will be updated each year with respect to inflation (around 22 billion euro in 2023).

GFA is paid monthly to all families for each dependent child, from the seventh month of pregnancy to the 18th birthday and, if the child does not work and with a reduced amount, up to the 21st birthday. Furthermore, for each disabled dependent child, GFA is recognized without age limits. GFA is paid for each child, regardless of the working status of the parents (not employed, unemployed, recipients of basic income, employees, self-employed workers, freelancers and pensioners) and without income limits. For poor parents receiving UBI, the quota that was assigned for children has been replaced by the GFA at a higher amount.

To receive GFA, parents must pay taxes (if any) in Italy, and have been continuously resident in Italy for at least 2 years or must be the holder of an employment contract with a duration of at least 6 months. These limitations are designed to limit so-called ‘welfare shopping’, that is, a movement to Italy to receive GFA. Non-EU citizens must hold a residence permit; however, unreasonable limitations for non-citizens have been avoided, such as the 10 years of continuous residence required to access the UBI.

The amount disbursed is not the same for everyone, but is commensurate with the ISEE, an indicator of the economic condition of the household that takes into account both the income and wealth of both parents (even if not living together). ISEE is used in Italy to graduate the tariffs for numerous public services (from nurseries to tuition fees). For receipt of the GFA, the ISEE thresholds have been set, so that around 50% of families obtain the maximum (€175 per month in 2022). As ISEE increases beyond the bottom 50%, the amount is gradually reduced falling eventually to €50 per month for the top 20% of wealthiest families. These amounts are slightly increased (between €85 and €15, declining with the ISEE) for children above the second order. Furthermore, families with four or more children receive a flat rate of €100 per month. Finally, to encourage the work of both parents, GFA is increased by €30 per month per child if both parents work, for those who receive the maximum allowance, reducing to zero for those receiving the minimum allowance.

Beyond the aforementioned inflation adjustment, these amounts have been partially increased by the new Conservative government: from 1 January 2023, the GFA increases by 50% for everyone during the child’s first year of life, regardless of the GFA level. Furthermore, with the exclusion of very wealthy families, for children from the third order onwards, the GFA increases by a further 50% up to the third year of age.

In summary, GFA is *efficient,* because it summarizes all the monetary transfers for families with dependent children under 21 in a single measure, thus greatly simplifying the regulatory framework (Renga, 2022, Part IV, Chapter 3). Furthermore, GFA is *universal,* because all children have the right to receive some benefit. Finally, GFA is *inversely proportional to wealth and income*. The payments are set at the maximum level for about the bottom half of all children and at the minimum level for about the top 20% of all children, with intermediate payments to those between these levels.

It is, therefore, a law that seeks above all to guarantee horizontal equity, i.e., a basic monetary support for all children. However, the GFA also pursues vertical equity goals; since for the richest 50% of households, the amounts disbursed decrease as wealth and income grow.

### GFA and poverty

Even if the main purpose of the GFA is to combat low birth rates, the way it is designed could also make it effective in alleviating child poverty. It is also possible that the main purpose is partly achieved through the secondary one, because—as we will see—for less well-off couples the amount of the GFA covers a high proportion of the cost of a child. The pro-natalist effect of the GFA manifests especially for poorer couples. It is, therefore, useful—in reflecting on the possible effects of GFA on the condition and behavior of couples—to start from its possible effects on poverty levels. Scholars and public research institutes have focused precisely on these aspects, pressed by legislators and stakeholders worried by the fact that such radical legislative changes could penalize particular groups of families.

At the time of writing (at the beginning of 2023, 1 year after the GFA went into effect), it is still too early to analyze the effect of GFA on poverty. This will not be a trivial undertaking, because the measure has been implemented in 2022 together with non-marginal changes to the tax levy and measures to counter the increase in the price of electricity and gas bills, mostly in favor of less well-off families. However, some public agencies and some scholars, using various simulation techniques, have tried to measure the specific redistributive effects of GFA (Baldini et al., 2021; Biagetti et al., 2022; Istat, 2022b; Pacifico, 2021; Ufficio Parlamentare di Bilancio, 2022). In general, these models appear to be reliable. For example, the ISEE threshold below which a family receives the full allowance (€175 per month in 2022) was determined a priori based on the results of some of these simulations. With the aim of benefiting about half of the families with children, €175 was the amount that achieved this result in the simulations.

All the quoted models show that poverty should be reduced by GFA, and that the number of households that gain is more numerous than those that lose. We report some of the results of an Istat study (2022b) based on a micro-simulation model. The risk of relative poverty (here defined as the percentage of people in households with an equivalent income lower than 60% of the median income) decreases, going from 18.6% to 17.2%. Poverty reduction varies according to age and household type (Table 2). The favored categories are obviously those affected by the GFA: the youngest ages (0–14 and 15–24) and people aged 35–44, the age group with the most parents having children eligible for the GFA. The relative poverty increases for couples without children and those aged 65 +.

**Table 2:** Poverty risk (*) of individuals in Italy in 2022 by age and family type: an Istat simulation

	Without GFA (A)	With GFA (B)	Difference (B)–(A)
Age group			
0–14	25.2	21.4	− 3.8
15–24	25.0	22.5	− 2.5
25–34	18.9	17.8	− 1.1
35–44	21.6	19.2	− 2.4
45–64	17.9	17.0	− 0.9
65 +	11.7	11.9	0.1
Individuals by household type			
Single up to 64	24.3	24.3	0.0
Single 65 +	14.0	14.0	0.0
Couple without children, she up to 64	11.7	11.9	0.1
Couple without children, she 65 +	8.4	8.8	0.4
Couple with at least one child 0–17	24.1	20.3	− 3.7
Couple with only children 18 +	14.8	14.5	− 0.4
Single parent with at least one child 0–17	33.1	29.9	− 3.2
Single parent with only children 18 +	15.0	15.0	− 0.1
Other	21.6	21.4	− 0.2
Total	18.6	17.2	− 1.4

*The percentage of people in families with an equivalent income lower than 60% of the median income

These estimates are obtained with the Istat Fa-MiMod microsimulation model. The model makes it possible to replicate the functioning of the tax transfer system for a representative sample of Italian families. FaMiMod is based on matching of administrative data from the Ministry of Finance, INPS (The National Institute for Social Security) and the Istat survey on income and living conditions (Eu-Silc)

Source: Istat (2022b)

In the same report, Istat also indicates the percentage of households that gain and lose with the introduction of GFA, according to the level of family income, without considering the 100% safeguard clause of the first year that ensures there are no losers in 2022 (Table 3). For two-thirds of all households, income would have been roughly the same following the reform as it would have been prior to the reform, while 24% of households would have gained and 9% would have lost. The percentages of both winners and losers are higher in the lower income classes. This includes both the unemployed (who previously received nothing for children) and low-income employees who received the maximum of both family allowances and tax deductions and, if they had a low income and 3 + children, also €145 per month based on Law 448/1998. As income increases, the percentage of winners and losers decreases. While for the richest quintile, GFA is low (€50 per month per child), for these families before the reform, family allowances and tax deductions were also very low. Finally, for all quintiles other than the richest quintile, the average change in income of the winners is greater than the average change in income of the losers.

**Table 3:** Winners and the losers of GFA by household income quintile in 2022: an Istat simulation

Quintile, (lowest to highest)	Winners			Losers		
	Monthly average gain €	Average change in Income %	% of households	Monthly average lost €	Average change in income %	% of households
First	+ 159	+ 7.7	30	− 63	− 4.1	16
Second	+ 174	+ 5.5	29	− 29	− 1.2	17
Third	+ 162	+ 4.0	25	− 47	− 1.5	8
Forth	+ 114	+ 2.3	24	− 74	− 1.8	4
Fifth	+ 71	+ 0.9	15	− 79	− 1.0	2
Total	143	+ 3.5	24	− 49	− 1.8	9

Note and source: see Table 2

Istat did not detail these calculations according to the number of children for the different levels of household income. Looking at other simulations, the results are unclear. According to the Ufficio Parlamentare di Bilancio (2022), GFA brings higher benefits for large families. With the new allowance, a single-income family headed by an employee with four children and an ISEE under the median, should receive monthly around 142€ more per child than in the previous situation. The benefit per child drops to 104€, 92€, and 83€, respectively, for the same type of family with three, two and one child. On the other hand, Biagetti et al. (2022) and Pacifico (2021) show that the measure would be able to significantly reduce poverty for families with one or two minor children, but not for all families with three children or more.

After decades of increasing poverty, the data on poverty over the coming years will allow us to confirm whether and to what extent the economic situation of Italian and foreign children and their parents has improved. However, the results of the simulations in Table 3 suggest that some changes should be introduced to eliminate the losses that would be incurred by 16–17% of low-income households. The safeguard against losses applied to 100% households in 2022, according to the law, drops to 67% in 2023 and then to 33% until February 2025 after which it is eliminated.

The simulations of the impact of GFA on poverty assume that there are no changes in the composition of the simulated household. A change in the composition of the household would occur if another child was born into the household. While the new-born child would attract a higher household GFA payment, the additional income would be less than the cost of the child and the poverty status of the family may worsen. Thus, poverty alleviation and pronatalist objectives might conflict with each other. Will Italian families use the additional income from GFA to increase their living standard or will they use it to help finance an additional child? This question is addressed in the following section.

### GFA and births

The effects of GFA on fertility can only be measured in years to come. For now, it is only possible to make hypotheses based on results already obtained for Italy on the effect of monetary transfers on births, on some recent estimates of the costs of a child, and on a brief analysis of the current characteristics of Italian fertility and services dedicated to families with children.

Above, we have cited studies that have shown how the increase in monetary transfers to low- or middle-income families in Italy have favored an increase in fertility and a reduction of abortion precisely for the categories involved in the intervention, while the behaviors of couples not benefiting did not change (Billari et al., 2005; Boccuzzo et al., 2008). A similar, more recent result showed an increase in fertility for poor couples benefiting from the UBI (Dachille & De Paola, 2022).

It is possible that these results could also be repeated for GFA, which for less well-off families covers a substantial part of the cost of an (extra) child. According to calculations based on the Istat consumption survey, the direct monthly consumption for a minor child in an Italian family was €650 in 2019, but with substantial differences according to family income, from €300 for the poorest quintile to €800 for the richest (Bovini & Colonna, 2021; Bank of Italy 2022, 60–69). Adopting these estimates, for the poorest fifth of families GFA (€175) covers more than half of a child’s direct consumption, while as income increases, the relative contribution of GFA decreases, becoming almost irrelevant for the richest quintile, where GFA is only €50 a month. However, these differences, absolute and relative, were similar in the previous tax transfer regime. For example, in the city of Milan in 2014, public transfers in favor of a 10-year-old child covered 40% of the cost of the child for a couple with 40% of the average income, 25% for a couple with an income equal to the average, 20% for a couple with income 50% above the average (Penne et al., 2019).

Bovini and Colonna (2021) also show that, for families in absolute poverty, direct monthly consumption is even lower, around €200 for each minor child. For them, GFA would almost cover the entire cost of the child. The direct costs for a minor child of a poor family can be this low, because in Italy, healthcare is free for everyone, as well as education for those aged 6–18 years. Moreover, public nurseries and kindergartens, and public universities are also cheap for families with low ISEE. Finally, in many poor families, only one parent works, and, therefore, the other (almost always the mother) stays at home to look after the children or works part-time, so care activity can be an opportunity cost, but it is not a direct cost. For poor families, two-thirds of direct consumption for each child goes into food and his/her share of rent and utility bills. For the other items (clothes, shoes, education, health, transport, culture, sport, free time and more), the monthly expenditure for a minor child of a family in absolute poverty is €60, against €400 or more for non-poor families.

The effect of a birth—especially for families with medium to low incomes—is consistent across families with different characteristics, as GFA does not depend on the condition of the parents, but only on the existence of the child. The uncertainty component linked to the previous measures (family allowances, deductions and episodic bonuses) is eliminated: the only thing that can change is the monetary entity of GFA, which, growing as income and wealth of the family decrease, can be seen as a kind of insurance to guarantee a certain base-resource for childrearing. From this point of view, GFA reduces the economic uncertainty that restrains both the intentions and the achievements of fertility, in Italy, as in other developed countries (Fahlén & Oláh, 2018; Vignoli et al., 2020).

Having said this, it is unlikely that this single policy change could be the panacea to reverse 40 years of low fertility in Italy, for at least two reasons.

The first reason is that there has been a recent structural change in Italian fertility. Until the end of the twentieth century, fertility decline in Italy was due to falling numbers of families having three or more children. While Italian women born in 1940 had an average of 2.16 children, those born in 1955 had 1.83 children, with 75% of this decline being explained by the decrease in parities of three or more. The probability of having the first child actually increased across these cohorts (Zeman et al., 2018). For cohorts born around 1955, the probability of having the first or the second child was, in fact, quite similar for Italian and French women, whereas the lower general fertility in Italy was mainly due to the lack of children of higher parities, and the period low fertility of the 1990s was due mainly to a tempo or timing effect (Sobotka, 2004).

However, during the first two decades of the twenty-first century, this situation has changed drastically. Italian low fertility is today mostly the result of a high proportion of men and women having no children. Italian women born in 1970 had 1.49 children, compared with 1.83 for the 1955 cohort, with 71% of this decline due to the fall in first-born children. The contribution to the TFR of births of orders higher than the first has, meanwhile, remained constant (Zeman et al., 2018). According to Istat (2022c), the proportion of childless Italian women rose from 10% for the 1950 cohort, to 12% for the 1960 cohort, 20% for the 1970 cohort, 25% for the 1980 cohort (for the last cohort, fertility at ages 40–49 was estimated).

For the large majority of these women, this was not a choice but was the result of the concomitance of various constraining factors (Tanturri & Mencarini, 2008). Today, the “delay syndrome” of a deferral of family formation is even more pronounced than in the first two decades of Italian low fertility (Livi Bacci, 2001). Moreover, the decline in fertility after 2012 is entirely among the decreasing proportion of young women (and men) without a co-residing partner, while the propensity to have children within couples is constant (Table 4).

**Table 4:** Couple status of woman and fertility of women in a co-residing heterosexual couple in Italy during 2012–20, by year and age*

	Age 18–34		Age 35–49	
	% In a couple	Fertility of women in a couple**	% In a couple	Fertility of women in a couple**
2012	36.7	0.205	76.1	0.049
2013	35.6	0.212	75.2	0.047
2014	34.2	0.199	75.1	0.045
2015	33.2	0.195	74.9	0.049
2016	33.1	0.207	74.8	0.048
2017	32.6	0.196	75.0	0.048
2018	32.6	0.210	74.9	0.051
2019	32.0	0.198	74.8	0.051
2020	31.3	0.205	75.0	0.047

*For about 1.2% of women a year it was not possible to define whether or not they were in a co-residing couple

**Proportion of women who had a child in the year of the interview or in the previous one

In the Italian Labor Survey data base, the two co-resident partners and the dyadic mother/child are identified by crossing the kinship relation code with the head of the household. The combined proportion of children for whom it was not possible to identify a co-resident mother and the proportion of women for whom it was not possible to understand whether or not they were living in a couple relationship make up less than 2% of the observations. The survey data show that the share of births to single mothers over the 2012–20 period in Italy remained constant at around 7% (roughly the same as in official data) and the probability of having a child was ten times higher for women in a co-resident couple than for women who did not live with a partner

Source: Italian Labor Survey, years 2012–2020

While GFA may help some co-resident couples to have an (extra) child, it is unlikely to increase the proportion of young women and men who live as a couple, an almost indispensable premise in the Italian context for having a newborn. This proportion will increase only if the high degree of uncertainty and the economic difficulties that hold back the formation of young couples are mitigated, in particular, the precariousness nature of employment, the high rents and the cost of house mortgages which, moreover, are granted in Italy only to couples where at least one of the two partners has a stable job (Vignoli et al., 2016).

 GFA is designed to alleviate the direct costs of children, not the indirect costs. This is the second reason that it is unlikely to affect fertility in Italy. GFA does not address the conciliation of market work and childcare (Rosina & Luppi, 2022). In Italy today, the probability of having a first and second child is higher for couples, where both partners have stable jobs (Dalla Zuanna et al., 2022). Childcare and paid work can be reconciled if couples have easy access to cheap childcare services. In Italy, nurseries are expensive (with the exception of public services for poor couples) and the supply of childcare centers is deficient in large areas of the country. There is also a lack of alternative services, such as child-minders or public baby-sitters. Furthermore, although public primary and secondary schools (ages 6–18) are cheap and cover 90% of Italian students, most schools end at around 1.00 p.m, leaving the parents with the tasks of preparing lunch and caring for the children in the afternoon, conflicting with normal working hours. Fully paid paternity leave is also very short in Italy being just 10 days at the end of 2022, and optional leave (paid only at 30%) is rarely used by fathers (Del Boca, 2015). From 2023, the new government has increased parental leave (paid at 80%) by 1 month and it can be taken either by mothers or fathers. This is a signal on a road to gender equality, but it is less supportive than policies implemented by other European low fertility countries, such as Spain and Finland (European Parliament, 2022).

Finally, Italy is one of the European countries, where the level of equity in sharing of childcare responsibilities between the mother and the father is low (Pailhé et al., 2019). Among Italian couples where this gender imbalance is less accentuated, the probability of having an extra child is higher (Cooke, 2009).

## Conclusion

The GFA has the great merit of having ordered and modernized monetary transfers in favor of families with children in Italy, covering large groups of families that were previously excluded from full benefits. Its main objective would be to increase the birth rate, but—due to the way it was designed—it is likely that its potential effect on fertility will be limited to couples with modest incomes. The direct cost of children for middle to high income couples is so high that it is unthinkable that the state could cover it to a significant extent, given the conditions of Italian public finance, which make very unlikely a significant increase in the amounts—in the coming years—for less poor families. Moreover, further studies might take into consideration a more integrated approach on the existing policies (to support income, fertility and providing services) to study the combined effect on the reduction of the children-related costs and the poverty risk for families with children.

For Italian fertility to increase in a stable and generalized way, wider-ranging policies are required to change the general conditions of young people of reproductive age (Castiglioni & Dalla-Zuanna, 2017, chap. 7). Youth employment insecurity should be lowered, the employment and income of young fathers and, more especially, young mothers needs to increase, young couples need to be helped to set up homes, net wages should grow, especially in the early years of working life. Furthermore, the combination of childcare and paid work should be reconciled by reducing the costs of public and private childcare at ages 0–5 and by radically reforming the didactical organization and timetables of primary and secondary schools. Last but not least, both general and focused policies should favor fathers involvement in childcare and homecare, accelerating a cultural switch towards the reduction of gender bias among couples, both in unpaid and paid work.

## Data Availability

Not applicable.
